# Factors associated with late presentation for HIV care in a single Belgian reference center: 2006–2017

**DOI:** 10.1038/s41598-018-26852-0

**Published:** 2018-06-05

**Authors:** Gilles Darcis, Iseult Lambert, Anne-Sophie Sauvage, Frédéric Frippiat, Christelle Meuris, Françoise Uurlings, Marianne Lecomte, Philippe Léonard, Jean-Baptiste Giot, Karine Fombellida, Dolores Vaira, Michel Moutschen

**Affiliations:** 1 Infectious Diseases department, Liège University Hospital, Liège, Belgium; 2 AIDS Reference Laboratory, Liège University, Liège, Belgium

## Abstract

Late presentation for HIV care is a major issue and the cause of higher morbidity, mortality and transmission. In this regard, we analyzed the characteristics of patients presenting for care at our center from January 2006 to July 2017 (n = 687). The majority of the studied population was of African origin (54.3%) with heterosexual women representing the main group (n = 292; 42.5%). 44% of the patients were late presenters (LP) (presenting for care with CD4 T cells <350/mm^3^ or an AIDS defining event) and 24% were late presenters with advanced disease (LP-AD) (presenting for care with CD4 T cells <200/mm^3^ or an AIDS defining event). A very high risk of being LP and LP-AD was associated with Sub-Saharan origin (OR 3.4 and 2.6 respectively). Other factors independently associated with LP or LP-AD were age (OR 1.3), male gender (OR 2.0 and 1.5 respectively) and heterosexual route of transmission (OR 2.4 and 2.3 respectively). A significant increase in HIV screening without forgetting those groups would contribute to earlier HIV diagnosis, a key element to end the HIV epidemic. To achieve this goal, addressing the specific hurdles to HIV testing in the migrant population is critical.

## Introduction

Since a few years, the 90-90-90 target has become a central column of the worldwide pursuit to end the AIDS epidemic. The target reflects a fundamental shift in the world’s approach to HIV treatment towards the importance of maximizing viral suppression among people living with HIV^1^. Antiretroviral therapy (ART) is now recommended for all those with HIV infection in order to prevent AIDS-related illness and to decrease the risk of transmission.

Late presentation (LP) for care is a significant and persistent issue for the success of ART outcomes. Indeed, late presenters have an extended chance to transmit HIV while they are unaware of their HIV infection^2–5^. LP is also associated with a negative impact on mortality and morbidity^6,7^. Regarding to immunological recovery, patients who began ART with ≤350 CD4 T cells/mm^3^ generally do not regain normal CD4 naïve/memory T cell ratios^8^. The START trial clearly showed that the immediate initiation of antiretroviral therapy was superior to deferral of such therapy until the CD4 T cell count declined to 350 cells/mm^3,9^. A beneficial effect of immediate antiretroviral therapy was evident for both serious AIDS-related and serious non–AIDS-related events^9^, and the relative risk reduction was greatest among older participants, those with high plasma HIV RNA viral loads and with low baseline CD4 to CD8 ratios^10^.

Moreover, early initiation of ART restricts the establishment of the HIV reservoirs that are considered as a major barrier to the cure of HIV infection^11,12^. Early treatment not only reduces the size of the latent reservoir, but also alters the composition of the reservoir in a way that may enhance the efficacy of potential eradication therapies^13^. Indeed, unless ART is started early, a large majority of latent viruses which constitute the HIV reservoir carries mutations that render infected cells insensitive to cytotoxic T lymphocytes (CTL)^13^.

Because of its many negative consequences for infected individuals and HIV transmission, it is critical to identify factors associated with late presentation for HIV care. The identification of those factors could indeed improve the effectiveness of HIV testing strategies with a focus on susceptible and possibly neglected groups.

A cross-European update from 34 countries on the prevalence and factors associated with LP has recently been published^14^. The huge number of patients included in this study allowed for an interesting and statistically significant analysis of factors associated with LP overtime. However, it highlighted also that there are major differences between European regions, reducing the usefulness of such an analysis as the aim is to adapt the local testing strategies.

In this study, we conducted a rigorous analysis of factors associated with LP and LP with advanced disease (LP-AD) in a single Belgian reference center, with the ultimate objective to improve local early screening policies as well as in countries or regions around the world sharing similar epidemiologic and demographic characteristics.

## Methods

### Patients and definition of late presentation

This was a retrospective cohort study. People aged at least 16 who presented for care (first clinic visit) at the Liege University Hospital (Belgium) between the 1^st^ January 2006 and 31 July 2017 were included in the study. Patients who had already received care for their HIV infection at another clinical center were excluded from the study as well as people already treated for more than one month before the first clinic visit. According to the consensus definition of LP^15^, patients who were previously diagnosed but who did not access to care were kept in the analysis. Indeed, the term ‘presentation for care’ means attendance at a health care facility that is able to monitor progression of HIV infection and initiate appropriate medical care. Diagnosis of HIV infection means presentation for care only if diagnosis of HIV infection is linked to appropriate access to care^15^. This information was obtained based on standardized procedure used to determine medical histories at first presentation for care. Although it is not completely excluded that some patients presented for care in another center at an earlier time, we believe this approach is an effective and rigorous way to be in line with the LP definition^15^.

LP was defined as presentation for care with CD4 T cells <350/mm^3^ or an AIDS defining event (at any CD4 T cell count) in the six months following first visit. LP with advanced HIV disease (LP-AD) was defined as presentation for care with CD4 T cells <200/mm^3^ or an AIDS defining event (at any CD4 T cell count) in the six months following first visit. Seroconverters (negative HIV test over the past six months; n = 10) with CD4 T cell count <350/mm^3^ were moved from the LP or LP-AD groups to the non-LP group. By definition, such people are diagnosed soon after HIV infection, even if they have a low CD4 T cell count at HIV diagnosis. They would not have been reached by interventions that promote earlier targeted screening. Patients infected with HIV-2 were excluded from the analyses as kinetics of CD4 T cell count decrease might be different.

Information obtained at first clinic visit included date of birth, gender and geographical origin categorized as Belgium, Sub-Saharan Africa (SSA) and other to reflect the main group of immigrants in Belgium. HIV exposure group categorized as homo- bisexual, heterosexual, and other (including intravenous drug use (IDU)) was also collected. Participants with both sexual and IDU transmission risk (n = 5) were included in one of the sexual transmission groups.

Reported information finally included the screening context categorized as voluntary screening, medical condition, refugee (defined as a person fleeing armed conflict or persecution and asking or benefiting from assistance by the state, the Red Cross or other organizations), incidental screening (for instance before a blood donation), pregnancy, HIV^+^ partner or other/unknown. Information regarding AIDS-defining illnesses and CD4 T cell count at first entry into care at the HIV clinic was available and was included in the analyses.

The Ethical Committee of the University Hospital of Liège approved the study protocol (reference 2018/118). Patients were informed of data collection by their treating physician and patients could object to further collection of clinical data according to an opt-out procedure. All patients included were assigned unique identification numbers to anonymize the data and protect confidentiality. All methods were carried out in accordance with relevant guidelines and regulations^16^.

### Statistical analysis

Data were summarized as median and interquartile range (IQR) and extreme values for quantitative variables while frequency tables were used for the categorical findings. Multiple logistic regression was used to identify the impact of the patients’ characteristics on late presentation. Multiple imputation was used to replace missing values. Results were considered significant at the 5% critical level (p < 0.05). Data analysis was carried out using SAS (version 9.4 for Windows). R (version 3.2.5) packages were used for the figures.

## Results

Of 759 patients who presented for care at the Liege University Hospital (Belgium) between the 1^st^ January 2006 and 31 July 2017, 68 were excluded because there was evidence that these people had been previously followed at another center or treated before. Patients infected with HIV-2 (n = 4) were excluded from the analyses (see Methods). The characteristics of the 687 remaining individuals who first presented for care are shown in Table 1. The median age is 34 years. 44.7% are female. The majority of the studied population is of African origin (54.3%). Heterosexual transmission was reported in 61.0% of the participants.

**Table 1:** Characteristics of HIV patients included in the study, January 2006 to July 2017 (n = 687).

	N (%)	Median (IQR)	Extremes
Age (years)	687	34.0 (27.0–42.0)	16.0–68.0
Gender (Female)	307 (44.7)		
Origin			
Belgium	238 (34.6)		
SSA	373 (54.3)		
Others	76 (11.1)		
Mode of acquisition			
Heterosexual transmission	419 (61.0)		
Homo/Bisexual transmission	209 (30.4)		
Others or unknown	59 (8.6)		
First CD4 T cell count			
Number/mm³	687	386 (210–541)	0–1772
<200/mm³	155 (22.6)		
200–350/mm³	150 (21.8)		
>350/mm³	382 (55.6)		
Context of the screening			
Incidental screening	45 (6.6)		
Voluntary screening	72 (10.5)		
Pregnancy	20 (2.9)		
HIV-positive partner	45 (6.6)		
Medical problem	198 (28.8)		
Refugee	225 (32.8)		
Unknown	82 (11.9)		

SSA: Sub-Saharan Africa.

SD: standard deviation.

IQR: Interquartile range.

Among our 687 patient cohort, heterosexual women from SSA represented the main group (n = 292; 42.5%), which corresponds to the large majority of female patients (95.1%) and the heterosexual group (69.7%).

208 patients (30.3%) were Belgian men who have sex with men (MSM). Those patients represented nearly all the patients included in the homo/bisexual transmission group.

As indicated in Table 2, 44.0% of the patients were late presenters and 24.0% were late presenters with advanced disease. The variability of the LP and LP-AD percentages from one year to another, and according to the Belgian or migrant status, was not statistically significant (Fig. S1). In particular, there was no diminution of those percentages overtime. Supplementary Table 1 indicates the percentages of LP and LP-AD according to patients’ characteristics. The percentages of LP and LP-AD were higher in the groups of older individuals, in the female population, in the group of patients originating from SSA, in the heterosexual transmission group as well as in the group of refugees. In contrast, those percentages were lower in the patients diagnosed following a voluntary screening.

**Table 2:** Proportion of late presenters (LP) and late presenters with advanced disease (LP-AD) (N = 687).

	N (%)
Non LP	385 (56.0)
LP^(a)^	302 (44.0)
LP-AD^(b)^	165 (24.0)

^(a)^CD4 T cells <350/mm^3^ or an AIDS defining event (at any CD4) in the six months following first visit.

^(b)^With CD4 T cells <200/mm^3^ or an AIDS defining event (at any CD4) in the six months following first visit.

### Factors independently associated with late presentation and late presentation with advanced disease

Multiple logistic regression was used to measure the impact of the various patients’ characteristics, independently of one another, on late presentation and late presentation with advanced disease for HIV care.

Figure S2 and Table 3 present the adjusted odds ratios of being LP. Age (OR 1.3 (95% CI 1.1–1.5)), male gender (OR 2.0 (95% CI 1.3–3.1)), Sub-Saharan origin (OR 3.4 (95% CI 1.9–5.9)) as well as heterosexual route of transmission (OR 2.4 (95% CI 1.4–4.1)) are factors associated with a statistically significant higher risk of being LP. Migrants from other parts of the world than SSA had also a higher risk of being LP (OR 1.9 (95% CI 1.0–3.4)). Refugees or individuals whose HIV diagnosis was made in the context of a medical condition presented a modest risk of being LP which did not reach statistical relevance.

**Table 3:** Factors associated with late presentation (LP) – Multiple logistic regression (N = 687).

	Coefficient ± SE	Odds ratio (95% CI)	p-value
Intercept	21.6 ± 56.6	—	—
Year of presentation for care	−0.011 ± 0.028	1.0 (0.94–1.1)	0.69
**Age** (**by 10 years older**)	**0.23 ± 0.084**	**1.** **3** **(1.** **1–1.5)**	**0.0069**
**Sex** (**0** = **Female**, **1** = **Male**)	**0.35 ± 0.11**	**2.** **0** **(1.** **3–3.1)**	**0.0021**
**SSA**^a^ **origin** (**0** = **No**, **1** = **Yes**)	**0.** **61 ** **±** ** 0.** **14**	**3.** **4** **(1.** **9–5.9)**	**<0.0001**
**Other**^a^ **non belgian origin** (**0** = **No**, **1** = **Yes**)	**0.** **31 ** **±** ** 0.** **16**	**1.** **9** **(1.** **0–3.4)**	**0.044**
**Hetetosexual**^b^ **mode of acquisition** (**0** = **No**, **1** = **Yes**)	**0.** **43 ** **±** ** 0.** **14**	**2.** **4** **(1.** **4–4.1)**	**0.0024**
Other (non sexual) mode of acquisition (0 = No, 1 = Yes)	0.11 ± 0.26	1.2 (0.45–3.4)	0.68
Context of screening = Refugee (0 = No, 1 = Yes)	0.12 ± 0.12	1.3 (0.78–2.1)	0.34
Context of screening = Medical problem (0 = No, 1 = Yes)	0.17 ± 0.11	1.4 (0.90–2.2)	0.14
Context of screening = Voluntary screening (0 = No, 1 = Yes)	−0.036 ± 0.14	0.93 (0.53–1.6)	0.81

^a^Ref = Belgian.

^b^Ref = Homo/bisexual transmission.

SSA: Sub-Saharan Africa.

SE: standard error.

CI: confidence interval.

Bolded data are statistically relevant (p < 0.05).

Figure S3 and Table 4 present the adjusted odds ratios of being LP-AD. The same conclusions can be drawn for LP-AD as it did for LP. Indeed, Age (OR 1.3 (95% CI 1.1–1.5)), Sub-Saharan origin (OR 2.6 (95% CI 1.4–4.9)) as well as heterosexual route of transmission (OR 2.3 (95% CI 1.2–4.3)) were associated with a statistically relevant higher risk of being LP-AD. Migrants from other parts of the world than SSA had also a higher risk of being LP-AD (OR 2.3 (95% CI 1.1–4.8)).

**Table 4:** Factors associated with late presentation with advanced disease (LP-AD) – Multiple logistic regression (N = 687).

	Coefficient ± SE	Odds ratio (95%IC)	p-value
Intercept	11.5 ± 64.6	—	—
Year of presentation for care	−0.0066 ± 0.032	1.0 (0.93–1.1)	0.84
**Age** (**by 10 years older**)	**0.24 ± 0.095**	**1.3 (1.1–1.5)**	**0.011**
Sex (0 = Female, 1 = Male)	0.19 ± 0.12	1.5 (0.93–2.3)	0.10
**SSA**^a^ **origin** (**0** = **No**, **1** = **Yes**)	**0.47 ± 0.16**	**2.6 (1.4–4.9)**	**0.0036**
**Other**^a^ **non belgian origin** (**0** = **No**, **1** = **Yes**)	**0.42 ± 0.18**	**2.3 (1.1–4.8)**	**0.023**
**Hetetosexual**^b^ **mode of acquisition** (**0** = **No**, **1** = **Yes**)	**0.41 ± 0.16**	**2.3 (1.2–4.3)**	**0.013**
Other (non sexual) mode of acquisition (0 = No, 1 = Yes)	0.27 ± 0.30	1.7 (0.54–5.5)	0.36
Context of screening = Refugee (0 = No, 1 = Yes)	0.16 ± 0.14	1.4 (0.78–2.4)	0.27
**Context of screening** = **Medical problem** (**0** = **No**, **1** = **Yes**)	**0.38 ± 0.13**	**2.1 (1.3–3.6)**	**0.0062**
Context of screening = Voluntary screening (0 = No, 1 = Yes)	−0.11 ± 0.19	0.81 (0.38–1.7)	0.59

^a^Ref = Belgian.

^b^Ref = Homo/bisexual transmission.

SSA: Sub-Saharan Africa.

SE: standard error.

CI: confidence interval.

Bolded data are statistically relevant (p < 0.05).

Individuals whose HIV diagnosis was made in the context of a medical condition presented a higher risk of being LP-AD (OR 2.1 (95% CI 1.3–3.6)). Refugees and male presented a higher risk of being LP-AD (OR 1.4 (95% CI 0.78–2.4) and OR 1.5 (95% CI 0.93–2.3)) which did not reach statistical relevance, probably due to the sample size.

## Discussion

Current guidelines indicate that ART should always be recommended irrespective of the CD4 T cell count, but the lower the CD4 T count, the greater the urgency to start ART immediately^17^. The use of ART is recommended at any CD4 T cell count mainly in order to reduce sexual transmission, the risk of AIDS event and mother-to-child transmission of HIV^9^. Early ART has also the potential benefits of reducing the severity of acute symptoms, lowering the viral load set-point and HIV reservoir size, reducing viral genetic evolution and the establishment of CTL escape mutation, decreasing immune activation and chronic inflammation and preserving immune function^18,19^. Early ART could possibly enhance post-treatment control and response to future eradication strategies^20,21^. Consequently, the importance of early ART initiation has become increasingly evident in recent years.

In this context, analyzing the factors associated with a delayed presentation for care is certainly of importance. Several large studies have recently provided important data regarding to the risks of being LP and LP-AD^14,22^. However, these studies tend to erase the differences that exist between countries or regions and can potentially impact the factors associated with LP for HIV care. For instance, in our study, we showed that the majority (54.3%) of the studied population came from SSA. This particular characteristic of our cohort is certainly due to Belgian history, closely tied with several countries of SSA and in particular with Democratic Republic of Congo. In comparison, in a similar study recently performed in the neighboring Netherlands, the proportion on patients from SSA was only 15%^23^. In the COHERE cross-European study from 34 countries, the percentage of patients from SSA was only 8.7%^14^. Regional studies are thus needed in order to provide a more thorough analysis which will hopefully allow for a better HIV screening.

Of the 687 HIV patients included in our study between January 2006 and July 2017, 44% presented late and 24% were late presenters with advanced disease. These proportions are comparable to the estimates published by others^14,22–24^. We did not observe any diminution of those percentages over time, showing that the efforts made during the last ten years to improve and increase HIV testing were insufficient to significantly decrease the number of late presenters.

We showed that factors associated with LP and LP-AD were age, male gender, migrant status as well as heterosexual route of transmission. Factors associated with LP and LP-AD were similar, which makes sense since LP-AD constitutes a sub-group of LP. Those factors have also been reported by others^14,22–25^.

Given the large proportion of migrants from SSA in our cohort (54.3%) and the associated very high risk to be late presenters or late presenters with advanced disease (OR 3.4 (95% CI 1.9–5.9) and 2.6 (95% CI 1.4–4.9) respectively), this migrant population requires special attention. Although some patients in this group have acquired HIV in Africa and reached Belgium at an advanced stage of the disease, post-migration HIV acquisition is more frequent than previously thought. In a recent report by Alvarez-del Arco, the proportion of post-migration HIV acquisition within Europe was 63%^26^. The proportion of post-migration HIV acquisition was higher for Latin America and Caribbean migrants, and was also significant (45%) for people from SSA^26^. Migrant men who have sex with men appear at particular risk of HIV acquisition post-migration as supported by the frequent acquisition of viral clades not prevalent in native countries^27^. In our cohort however, the majority of patients from SSA were heterosexual women who represented 42.5% of the total studied population. Interestingly, in a French report by Desgrées-du-Loû and colleagues, it was estimated that at least 30% of HIV-infected women born in SSA acquired HIV while living in France^28^. More recently, Pannetier *et al*. showed that sub-Saharan African women living in the Paris region were regularly exposed to non-consensual sex (NCS) forced sex in their lifetimes^29^. They further provided evidence of an association between NCS after migration and post-migration acquisition of HIV among sub-Saharan African migrant women^29^. Those reports clearly highlight the need for primary prevention programs targeting these communities. Improving access to HIV testing and treatment for all infected persons, irrespective of their immigration status, could certainly impact on reducing incidence of late presentation and infection rates both within and beyond migrant communities. This would impose addressing the numerous hurdles to HIV screening and care in migrant communities, including cultural and linguistic barriers, racism and xenophobia as well as stigma from their communities^30^.

We further showed that age, male gender and heterosexual route of transmission are independent factors associated with LP and LP-AD. Indeed, people who are more likely to be diagnosed and to present late usually do not consider themselves as “risk categories”. HIV screening procedures basically depend on an individual’s perception of his/her HIV risk. For instance, MSM have a lower risk of being LP in our analysis, most likely because they better perceive the risk of being infected by HIV.

Additionally, healthcare providers may consider HIV testing less often in individuals who are not perceived as being at high risk of HIV such as older individuals or heterosexuals, leading to missed opportunities for HIV diagnosis. High rates of missed opportunities for HIV diagnosis have previously been reported, highlighting the ongoing need for physician education on HIV testing and clinical signs suggestive of HIV infection^31,32^. This is particularly relevant for people aged 50 years since this group would benefit most from immediate initiation of ART as recently shown in a post-hoc subgroup analysis of the START trial^10^.

Late presentation for HIV remains a key unresolved issue with severe consequences at the individual, societal and economic levels^33^. As underlined by our work, information campaigns and more effective HIV testing strategies are needed to shrink the inadmissibly high rates of late presentation for HIV care. All age and risk groups have to be targeted for HIV testing. Voluntary testing has to be encouraged through education campaigns targeting high prevalence groups but also neglected groups with a higher risk of LP. The education of healthcare providers should also emphasize the importance of a widespread HIV testing.

Most importantly in the context of a large population from SSA, it is critical to address the identified blocks to HIV testing and HIV care in migrant communities. This will hopefully result in improved treatment outcomes and may reduce HIV transmission, a necessary step forward a better control of AIDS epidemic.

## Electronic supplementary material

S1, S2 and S3 figures. S1 table
