# Supplementary material for: Factors associated with late presentation for HIV care in a single Belgian reference center: 2006–2017
Source: Sci Rep. 2018 Jun 5;8:8594. doi: 10.1038/s41598-018-26852-0 (PMC5988738; doi:10.1038/s41598-018-26852-0)
Supplement: Supplementary file 1 — S1, S2 and S3 figures. S1 table [file 41598_2018_26852_MOESM1_ESM.docx]

**Factors associated with late presentation for HIV care in a single Belgian reference center: 2006-2017.**

**Running title: HIV late presenters in a single Belgian reference center**

**Authors**

Gilles Darcis^1*^, Iseult Lambert^1^, Anne-Sophie Sauvage^1^, Frédéric Frippiat^1^, Christelle Meuris^1^, Françoise Uurlings^1^, Marianne Lecomte^1^, Philippe Léonard^1^, Jean-Baptiste Giot^1^, Karine Fombellida^1^, Dolores Vaira^2^ and Michel Moutschen^1,2^

**Affiliation:**

1 Infectious Diseases department, Liège University Hospital, Liège, Belgium

2 AIDS Reference Laboratory, Liège University, Liège, Belgium

**Supplementary information**

**S1 Figure:** Evolution of the proportions of late presentation (LP) (panel A) and late presentation with advanced disease overtime (LP-AD) (panel B) according the Belgian or migrant status (including patients from SSA); January 2006 to July 2017 (n=687).

**A.**


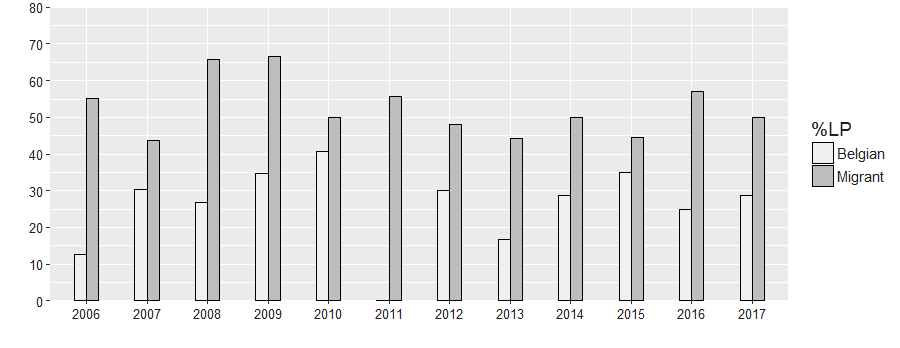


**B.**


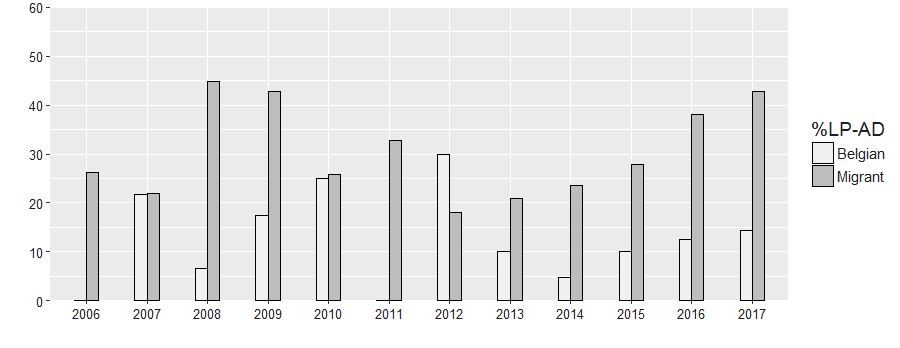


**S2 Figure.** Factors associated with late presentation for HIV patients (adjusted odds ratios)


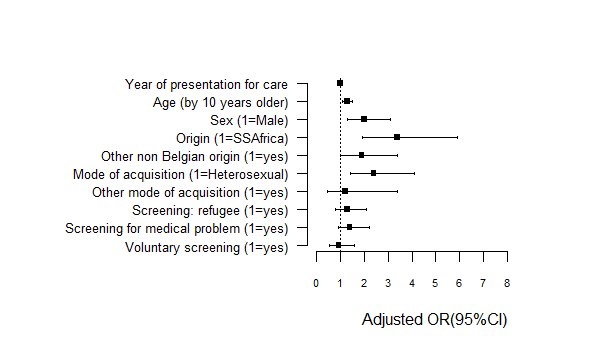


**S3 Figure.** Factors associated with for late presentation with advanced disease (adjusted odds ratios)


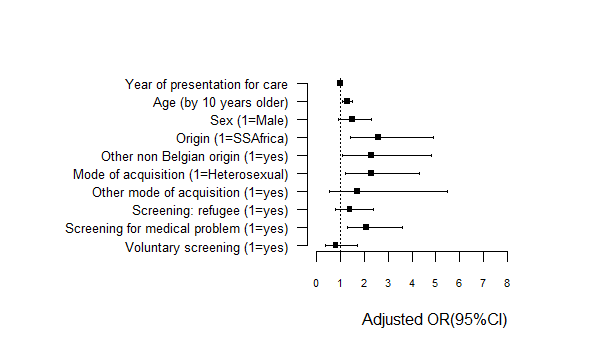


**S1 Table.** Percentages of late presenters (LP) and late presenters with advanced disease (LP-AD) according to patients’ characteristics.

|  | N | % LP | % LP-AD |
| --- | --- | --- | --- |
| Age |  |  |  |
| < 30 years | 226 | 36.3% | 16.8% |
| 30-50 | 385 | 48.6% | 27.3% |
| > 50 | 76 | 43.4% | 28.9% |
|  |  |  |  |
| Gender |  |  |  |
| Male | 380 | 40.3% | 21.8% |
| Female | 307 | 48.5% | 26.7% |
|  |  |  |  |
| Origin |  |  |  |
| Belgium | 238 | 26.9% | 13.9% |
| SSA | 373 | 55.5% | 30.0% |
| Other | 76 | 40.8% | 26.3% |
|  |  |  |  |
| Mode of acquisition |  |  |  |
| Heterosexual transmission | 419 | 53.2% | 30.1% |
| Homo/Bisexual transmission | 209 | 24.9% | 11.5% |
| Other | 21 | 42.9% | 28.6% |
|  |  |  |  |
| Context of the screening |  |  |  |
| Incidental screening | 45 | 40.0% | 22.2% |
| Voluntary screening | 72 | 26.4% | 8.3% |
| Pregnancy | 20 | 35.0% | 20.0% |
| HIV-positive partner | 45 | 37.8% | 15.6% |
| Medical problem | 198 | 40.9% | 26.8% |
| Refugee | 225 | 54.7% | 28.4% |

*SSA : Sub-Saharan Africa*
